# A multi-context learning approach for EEG epileptic seizure detection

**DOI:** 10.1186/s12918-018-0626-2

**Published:** 2018-11-22

**Authors:** Ye Yuan, Guangxu Xun, Kebin Jia, Aidong Zhang

**Affiliations:** 1 College of Information and Communication Engineering, Beijing University of Technology, Beijing, China; 2 Beijing Laboratory of Advanced Information Networks, Beijing University of Technology, Beijing, China; 3 Advanced Innovation Center for Future Internet Technology, Beijing University of Technology, Beijing, China; 4 Department of Computer Science and Engineering, State University of New York at Buffalo, Buffalo, USA

**Keywords:** Context learning, Deep learning, Epileptic seizure, Electroencephalogram, Feature extraction

## Abstract

**Background:**

Epilepsy is a neurological disease characterized by unprovoked seizures in the brain. The recent advances in sensor technologies allow researchers to analyze the collected biological records to improve the treatment of epilepsy. Electroencephalogram (EEG) is the most commonly used biological measurement to effectively capture the abnormalities of different brain areas during the EEG seizures. To avoid manual visual inspection from long-term EEG readings, automatic epileptic EEG seizure detection has become an important research issue in bioinformatics.

**Results:**

We present a multi-context learning approach to automatically detect EEG seizures by incorporating a feature fusion strategy. We generate EEG scalogram sequences from the EEG records by utilizing waveform transform to describe the frequency content over time. We propose a multi-stage unsupervised model that integrates the features extracted from the global handcrafted engineering, channel-wise deep learning, and EEG embeddings, respectively. The learned multi-context features are subsequently merged to train a seizure detector.

**Conclusions:**

To validate the effectiveness of the proposed approach, extensive experiments against several baseline methods are carried out on two benchmark biological datasets. The experimental results demonstrate that the representative context features from multiple perspectives can be learned by the proposed model, and further improve the performance for the task of EEG seizure detection.

## Background

Epilepsy is the fourth common neurological disease globally, and there are approximately 50 million people affected by epilepsy worldwide [1]. People with epilepsy are two to three times more likely to die prematurely compared to non-affected individuals [2]. Although anti-epileptic drugs are successful with certain individuals, about 30% of patients are unresponsive to such pharmacological intervention [3]. Epilepsy is characterized by unprovoked seizures associated with sudden irregular neuronal discharges in the brain [4]. In order to provide treatment and prevention to patients, epileptic seizure detection has garnered great interest among researchers in bioinformatics.

The recent advancement in sensor technologies has opened the possibility of closely monitoring patients’ conditions for a wide range of biomedical applications [5–7]. The biological data recorded by pervasive sensors can be used to analyze clinical observations of epileptic seizures, and thus improve the treatment of epilepsy [8]. In particular, the brain electrical activity can be effectively measured via electroencephalogram (EEG). For instance, multi-channel scalp EEG signal, a non-invasive biological measurement monitored by multiple EEG electrodes, is able to capture the abnormalities of different brain areas during the seizure. Unfortunately, long-term EEG visual inspection is extremely laborious for physicians, and requires highly-trained scarce neurological professionals to diagnose epilepsy [9]. This has motivated researchers to develop automatic EEG seizure detector using machine learning methodologies.

Most existing EEG seizure detectors can be regarded as a classification model containing four components: data acquisition, preprocessing, feature extraction, and classification [10]. Among these steps, feature extraction is key, since its aim is to characterize distinctive EEG patterns, which directly affect the performance of seizure detector. Consequently, on one hand, various handcrafted features have been employed to detect EEG seizures. Of the numerous available approaches, wavelet transform, an excellent tool for non-stationary and transient biological signal processing, stands out due to its effectiveness [11, 12]. Wavelet transform provides both time and frequency signal views simultaneously [13]. Not only can it be used for signal denoising, it can also extract the features with tiny variations and sudden changes that are difficult for physicians to observe. On the other hand, deep learning techniques have been adopted to automatically learn features from epileptic EEG signals [14, 15]. These deep learning-based methods have been proposed to capture seizure patterns from raw biological data by using multi-layer neural networks. Previous studies have validated that deep learning can achieve better detection performance than handcrafted feature engineering.

Despite many deep learning studies reporting promising results in EEG seizure detection, some challenges still need to be addressed. One of the major challenges is that most methods ignore the dynamic correlations between EEG timestamps and randomly feed each timestamp to the classifier. This leads to the failure of recognizing temporal signal patterns. Another challenge is the ambiguity of feature extraction. Since the EEG data always contains multiple channels, adopting conventional deep learning methods can hardly extract enough features for the task of EEG seizure detection [16]. Complementary information need to be extensively incorporated to enhance the feature representation.

In order to address the above challenges, we propose a multi-context seizure detection approach to unsupervisedly learn features of multi-channel EEG data from different perspectives. Specifically, we first utilize a fix-length sliding window to segment the entire EEG records into fragments, and adopt wavelet transform as preprocessing to express the fragment sequence in the time-frequency domain, depicted as EEG scalogram sequence. Taking the advantage of context learning in bioinformatics [17–19], we propose to incorporate handcrafted features to further capture representative patterns of EEG seizures. We summarize the main contributions of this paper as follows:

- We develop a channel-wise deep learning module to learn a dictionary of EEG scalogram fragments by unsupervisedly extracting inherent features from each EEG channel.
- We develop a embedding-based module, i.e., EEG embeddings, to learn temporal features from EEG scalogram sequence translated by the learned EEG dictionary.
- We propose a new multi-context fusion approach that explicitly incorporates the features extracted from the global handcrafted engineering, channel-wise deep learning, and EEG embeddings modules. The integrated features are subsequently used for EEG seizure detection.
- We empirically demonstrate that the proposed approach outperforms seven existing EEG seizure detection methods on two benchmark datasets.

The rest of the paper is organized as follows: The details of the proposed seizure detection approach are introduced in “Methods” section. Experimental results are presented and analyzed in “Results” section. “Discussion” section discusses the effectiveness of our model, and the study is concluded in “Conclusions” section.

## Methods

In this section, we present the overview of our EEG seizure detection approach, followed by detailed discussions of each part of the proposed model.

### Framework

**Fig. 1:**
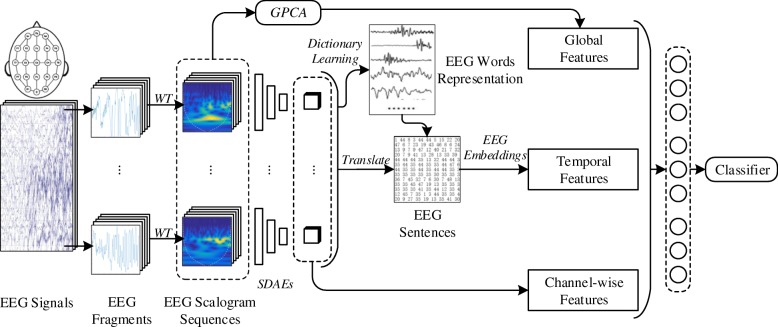
Schematic illustration of the overall approach pipeline. In this framework, we focus on extracting EEG context features in three aspects, referred to global, channel-wise, and temporal features, utilizing global principal component analysis (GPCA), stacked denoising autoencoders (SDAEs), and EEG embeddings, respectively. Then we feed the integrated features to the seizure detector

Figure 1 illustrates the framework of our proposed seizure detection model. Our approach aims at capturing latent seizure characteristics from EEG records in various aspects. Since the EEG records are time series and contain different physiological patterns in different intervals (i.e., timestamps) [20], we firstly segment and convert the entire EEG records into several EEG scalogram sequences using wavelet transform. Then we propose to extract EEG context features in three aspects, referred to as global, channel-wise, and temporal features, utilizing global principal component analysis (GPCA), stacked denoising autoencoders (SDAEs), and EEG embeddings, respectively. Finally, all the learned features are concatenated and fed to a support vector machine (SVM) classifier [21] for EEG seizure detection.

### EEG scalogram representation

Brain abnormality is often reflected by increased amplitudes and frequency changes in EEG signals [22]. Thus, incorporating signal processing knowledge into EEG seizure detection is able to enhance its performance. Wavelet transform enables us to represent each EEG fragment with an EEG scalogram in the time-frequency domain, making our model robust against signal shifting and noise over time. Formally, given a single-channel EEG fragment *x*(*t*), we can generate its scalogram using continuous wavelet (CWT) [13], as follows:

1 $$\begin{array}{@{}rcl@{}}  \begin{aligned} \text{scalogram}_{x}(a,\tau)= & |\text{CWT}_{x}(a,\tau)|^{2} \\ = & |\frac{1}{\sqrt{a}}{\int\nolimits}_{-\infty}^{\infty}x(t)\Psi^{\ast}\left(\frac{t-\tau}{a}\right)dt|^{2}, \end{aligned} \end{array} $$

where *Ψ* is the mother wavelet, and the asterisk denotes the function of complex conjugate. Here the dilation parameters *a* and *τ* in Eq. (1) determine the oscillatory frequency and shifting position of the wavelet, respectively. In this way, we can describe the time-varied frequency content in epileptic EEG signals, and further extract features using our proposed multi-context learning module. In our model, we employ Morlet, a commonly used mother wavelet, to generate EEG scalogram.

### EEG multi-context learning

The motivation of learning multi-context features arises from the inability of a single feature to reach accurate and robust performance. In particular, we attempt to unsupervisedly extract a set of abstract features from EEG scalogram sequences by incorporating the inter and intra correlations of EEG channels, as well as the dynamic relationships among EEG timestamps, namely global, channel-wise, and temporal features, respectively.

#### Principal component analysis for EEG global feature selection

To alleviate the influence of feature irrelevancy and redundancy, according to the handcrafted feature engineering, we adopt GPCA to derive top-*k* principal components of all-channel EEG scalograms, referred to the global features. The principal component number *k* is optimized by employing the leave-one-out validation [23]. In this way, we can exclude redundant and irrelevant information carried by each EEG channel to enhance the inter-channel representation.

#### Deep model for EEG channel-wise feature learning

Regarding the generated EEG scalograms, we take them as spectral images and separately extract their spatial features from each channel, referred to the channel-wise features. More specifically, the EEG scalogram fragments of each EEG channel are further processed through SDAEs [24] constructed by a series of denoising autoencoders (DAE) [25].

**Fig. 2:**
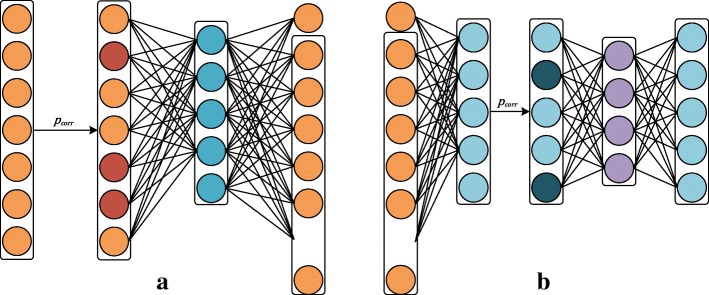
Deep model for EEG channel-wise feature learning. We separately extract spatial features of scalograms from each EEG channel. **a** represents the structure of DAE network and **b** represents the structure of SDAEs network

DAE is a neural network with one hidden layer, which can be expressed by learning an encoder network and a decoder network, as shown in Fig. 2a. In order to uncover robust hidden representations, different from the conventional autoencoder (AE) [26], DAE randomly corrupts input data $\hat {x}$ by sampling $\hat {x} \sim P_{corr}(\hat {x} \mid x)$ before the feature encoding. In our model, we assume that there are *C* channels of the input. Given the input vector of each channel *x*, we can obtain its reconstruct vector *y* by:

2 $$\begin{array}{@{}rcl@{}}  y = h_{W,b}(x) = f\left(W^{(l+1)}f\left(W^{(l)}\hat{x} + b^{(l)}\right) + b^{(l+1)}\right), \end{array} $$

where *b*^(*l*)^ and *W*^(*l*)^ are the learnable bias vector and weight matrix in the *l*-th layer, respectively. Here in Eq. (2), we use the sigmoid as the activation function defined as *f*(*z*)=1/(1+ exp(−*z*)). Subsequently, given an unlabeled training sample $x^{(i)} \in \mathbb {R}^{n}$, we use cross entropy to measure the reconstruction error between the input *x*^(*i*)^ and output *y*^(*i*)^, as follows:

$$\begin{array}{@{}rcl@{}} \begin{aligned} \mathcal{L}_{\text{DAE}}\left(x^{(i)},h_{W,b}\left(x^{(i)}\right)\right) =& - \sum\limits_{k=1}^{n}\left[x^{(i)}_{k}\log{\left(y^{(i)}_{k}\right)} \right. \\ & \left. + \left(1-x^{(i)}_{k}\right)\log{\left(1-y^{(i)}_{k}\right)}\right]. \end{aligned} \end{array} $$

By stacking DAE, we obtain a deep neural network, i.e., SDAEs, as shown in Fig. 2b. We adopt greedy layer-wise strategy [27] to train the SDAEs model. In particular, the output hidden features extracted from the previous layer of SDAEs is fed to the next layer as input. The learnable parameters of each layer is trained individually while keeping the parameters of the previous layers fixed. After the training, in our model, we combine all the channel features in the last hidden layer of SDAEs as the channel-wise features. These features are effective to represent the unique characteristics of each channel in a high-order vector space.

Furthermore, as the SDAEs is trained, we also obtain a dictionary of basic EEG scalogram patterns (i.e., EEG words), where each pattern corresponds to one hidden unit and can be represented as the one-hot index value of hidden unit. Since different activation values of hidden units reflect different word distributions, each EEG fragment can be then regarded as a weighted combination of EEG words contained in the learned EEG dictionary [18]. In this way, we can utilize a max probability pooling to sample (i.e., translate) the EEG fragment as an EEG word to further represent the main EEG pattern activated in this fragment. Consequently, a sequence of EEG scalograms can be translated into a sequence of EEG words, regarded as EEG sentence, shown in Fig. 1. This creates an interpretable bridge between signal processing and semantic learning, providing a different angle to analyze EEG signals.

#### EEG embeddings for temporal feature extraction

In the task of biosignal processing, previous studies have validated the effectiveness of using temporal features to represent raw EEG signals [17, 18]. In our model, we adopt a similar strategy to extract temporal features utilizing the translated EEG sentence, referred to EEG embeddings. The main idea of learning EEG embeddings is to represent each EEG word as a unique fixed length vector and predict the current EEG word based on its context words. In this step, EEG words with similar semantics would be mapped to close positions in the embedding space incorporating the context information [28].

**Fig. 3:**
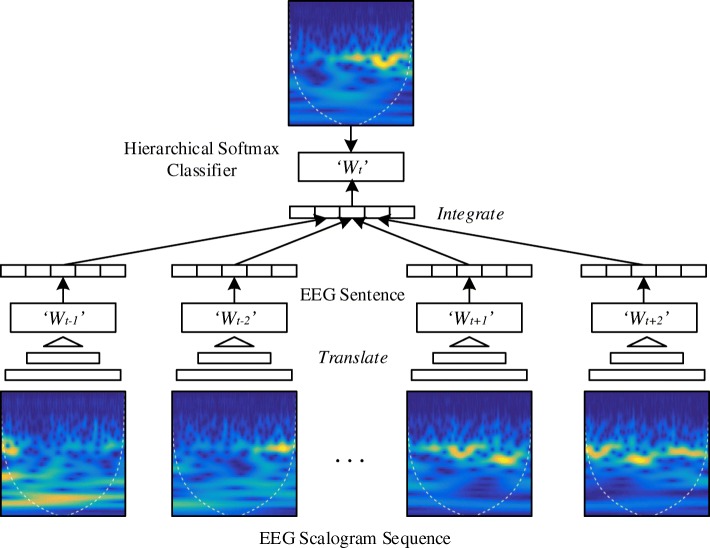
Framework of the EEG embeddings algorithm. We adopt a CBOW-based model to extract the temporal features from EEG scalogram sequences

Figure 3 illustrates the training step of EEG embeddings, where *w*_*t*_ denotes the current EEG word at timestamp *t*, and *w*_*t*−2_∼*w*_*t*+2_ denote the context EEG words at the previous 2 and the following 2 timestamps. Each EEG word *w*_*t*_ is mapped into a unique real-valued vector $v_{w_{t}} \in \mathbb {R}^{q}$, where *q* is the pre-defined dimensionality of EEG embeddings. Then, we use the softmax function to infer the current word *w*_*t*_ according to the integrated context word vectors.

Given an *T*-length EEG sentence {*w*_*t*_,*t*=1,2,...,*T*}, we define the objective function of EEG embeddings (EMB) by maximizing the average log probability to train the EEG embeddings, as follows:

$$\begin{array}{@{}rcl@{}} \begin{aligned} \mathcal{L}_{\text{EMB}} = & \frac{1}{T}\sum\limits_{t=k}^{T-k}\log p\left(w_{t} \mid w_{t-k}, w_{t-k+1}, \cdots, w_{t+k}\right) \\ = & \frac{1}{T}\sum\limits_{t=k}^{T-k}\log p\left(w_{t} \mid \text{ctx}\left(w_{t}\right)\right), \end{aligned} \end{array} $$

where *p*(*w*_*t*_∣ctx(*w*_*t*_)) denotes the prediction function that infers the current EEG word based on its context EEG words $\phantom {\dot {i}\!}\{v_{\text {ctx}(w_{t})}, t = 1,2,\cdots,T\}$.

Due to the large amount of context information, the training process of EEG embeddings is time consuming. To avoid this, we use a hierarchical structure to reduce the time complexity from *O*(*n*) to *O*(log*n*). More specifically, a hierarchical softmax function based on a binary Huffman tree is utilized. In a Huffman tree, the shortest path is assigned to the most frequent EEG word. Thus, our objective function can be further defined as:

3 $$\begin{array}{@{}rcl@{}}  p\left(w_{t} \mid \text{ctx}\left(w_{t}\right)\right)=\prod\limits_{j=2}^{l^{w_{t}}}p\left(d_{j}^{w_{t}} \mid \text{Intg}\left(w_{t}\right), \theta_{j-1}^{w_{t}}\right), \end{array} $$

where $d_{j}^{w_{t}} \in \{0,1\}$ is the Huffman code of word *w*_*t*_ in node *j*, and $\theta _{j-1}^{w_{t}}$ denotes the parameters of the sub-softmax functions on the Huffman tree path of word *w*_*t*_. Here the function Intg(·) in Eq. (3) denotes the integration of the context EEG word vectors, which is typically an average or a concatenation of the context vectors. Subsequently, the sub-softmax probability of hierarchical softmax function can be calculated as:

$$\begin{array}{@{}rcl@{}} \begin{aligned} p\left(d_{j}^{w_{t}}|\text{Intg}\left(w_{t}\right), \theta_{j-1}^{w_{t}}\right) &= \left[f\left(\left(\theta_{j-1}^{w_{t}}\right)^{T}\text{Intg}\left(w_{t}\right)\right)\right]^{1-d_{j}^{w_{t}}} \\ & \cdot \left[1-f\left(\left(\theta_{j-1}^{w_{t}}\right)^{T}\text{Intg}\left(w_{t}\right)\right)\right]^{d_{j}^{w_{t}}}. \end{aligned} \end{array} $$

The EEG embeddings can be trained with back-propagation. According to the constructing strategy of Huffman tree, more frequent EEG words are assigned shorter codes, and only the nodes on the path need to be updated for each training sample. This would effectively reduce the training complexity. After training all the EEG sentences, we can obtain a set of EEG embedding vectors with EEG semantic properties. These properties refer to the temporal relationship, since we incorporate the context information carried by the ordered EEG words in EEG sentence.

### Seizure detection using EEG multi-feature fusion

Based on the above learned multi-context features, we merge them together to derive a fusional hidden representation. Formally, given a training data *x*^(*i*)^, we can obtain the fusional feature of this sample as follows:

$$\begin{array}{@{}rcl@{}} x^{(i)}_{\text{Fusion}} = \left[x_{1}^{(i)} \oplus x_{2}^{(i)} \oplus \cdots \oplus x_{k}^{(i)}\right] \in \mathbb{R}^{\sum_{j=1}^{k} {n}_{j}}, \end{array} $$

where ⊕ denotes the concatenation operator, *k* is the feature index, and *n*_*j*_ denotes the dimensionality of each base feature. The integrated fusional vectors with the corresponding labels are then fed to train a seizure detector using SVM classifier [21]. Taking the advantages of multi-context features, SVM can learn a more distinct hyperplane to separate the non-ictal and ictal classes in the vector space.

## Results

To validate the performance of our proposed approach for EEG seizure detection, we conduct computational experiments on two benchmark datasets. After describing the datasets and our experiment settings, we briefly present quantitative results, to measure the quality of the features extracted by our proposed method.

### Datasets

In the experiments, two benchmark EEG datasets, named the CHB-MIT dataset and the Bonn dataset, are used for evaluation.

**Fig. 4:**
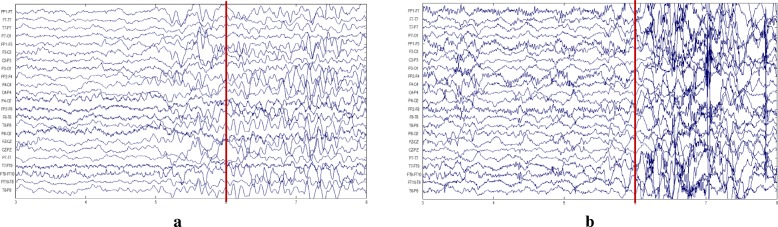
Two raw samples of multi-channel EEG signals on the CHB-MIT dataset. The red bar marks the beginning of EEG seizure, and both patients **a** and **b** start EEG seizure at the 6th timestamp

The CHB-MIT dataset is collected from the Children’s Hospital Boston [29]. This dataset is open access available and can be downloaded at the PhysioNet [30]. In this dataset, the multi-channel EEG signals are captured from 23 patients suffering from intractable seizures. Experts annotated the beginning and end of each seizure as ground truth. The EEG records consist of 23 channels, and the data of each channel is recorded at 256 Hz with 16-bit resolution. Figure 4 illustrates two examples of multi-channel EEG seizure onset within two different patients on the CHB-MIT dataset. Following the previous work [17], to enlarge the sample numbers, we generate 4302 23-channel EEG fragments from nine different patients by sliding a 3*sec* fix-length window with 1*sec* step length through the entire EEG signals.

**Fig. 5:**
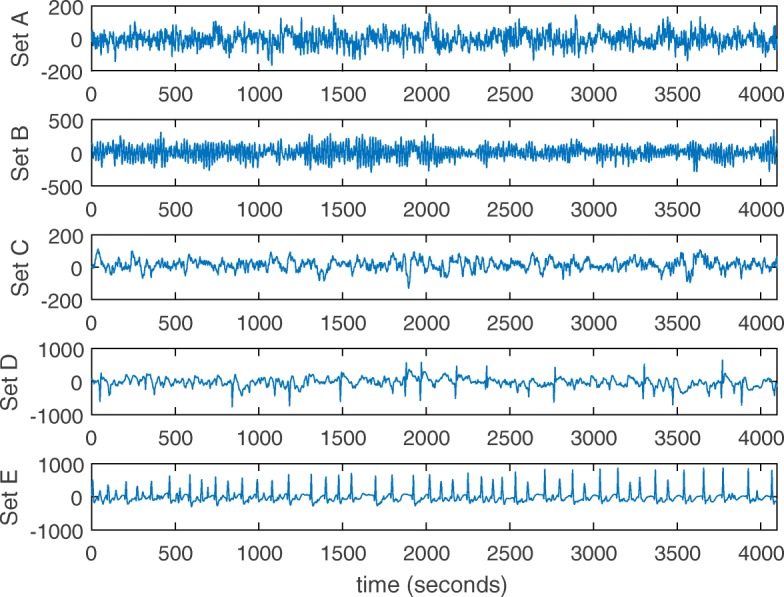
Four raw single-channel EEG samples on the Bonn dataset. Among all the subsets, only subset E contains epileptic seizure activity

The Bonn dataset is also a public dataset collected at the University of Bonn [31]. This dataset is categorized into 5 subsets (referred to A-E) according to expert visual inspection. Each subset contains 100 single-channel EEG signals of 23.6 s obtained from 5 patients. The EEG data is recorded at 173.61 Hz with 12-bit resolution. The raw EEG samples from sets A, B, C, D and E are shown in Fig. 5. Note that only subset E contains epileptic seizure activity. We adopt the same segmentation strategy and generate 10500 single-channel EEG fragments from all the subsets.

From the figures on the two datasets, we can observe that the EEG patterns are different among patients on both datasets, and the rhythms vary across channels unevenly and irregularly on the CHB-MIT dataset. This makes it difficult to detect EEG seizures from multi-channel records than the single-channel records.

### Experiment settings

In our experiment, each EEG fragment is labeled based on the ground truth as in one of the two classes: ictal and non-ictal states. Taking the computational expense into consideration, we adopt hold-out validation in the same way to [17, 32, 33]. Note that the holding-out portions of the dataset is a manner similar to cross-validation. In particular, we randomly divide the data to training and testing folds with ration 4:1. Due to the scarcity of abnormal events, we trim our experiment data to balance the number of ictal and non-ictal fragments. Furthermore, facing the high-dimensional inputs caused by multiple channels, we adopt 2-layer SDAEs for each EEG channel. We set 80 as the hidden size of the first layer and 60 for the second layer. The embedding size is fixed to be 20. Some training strategies including normalization and regularization are also utilized for our model.

*Evaluation metrics.* Since the seizure detection task belongs to a classification problem, we quantify the evaluation results according to the confusion matrix. Table 1 lists four different measurements used in our experiments, where TN, TP, FN, FP are true negative, true positive, false negative, and false positive, respectively. In addition, precision-recall (PR) and receiver operator characteristic (ROC) curves are plotted, respectively, to illustrate the quality of different seizure detectors. We also calculate the area-under-the-curve (AUC) of both two (i.e., AUC-PR and AUC-ROC), to measure the diagnostic ability of each method.

**Table 1:** Evaluation metrics definition

Measure	Definition
Precision	*TP*/(*TP*+*FP*)
Recall	*TP*/(*TP*+*FN*)
F1-score	2×*P*×*R*/(*P*+*R*)
Accuracy	(*TP*+*TN*)/(*TP*+*TN*+*FP*+*FN*)

*Baselines.* We employ several widely used classification algorithms as the baseline methods such as standard SVM [21], neural networks (NN) [34], and SDAEs [24]. For the sake of fairness, we employ principal component analysis (PCA) [23] as the data preprocessing mechanism for each method, referred to PSVM, PNN, and PSDAEs, respectively. We select top-*k* components with the same dimension of our proposed model. We also employ these methods in the time-frequency domain using wavelet transform, named WT-PSVM, WT-PNN, and WT-PSDAEs. Moreover, we compare the state-of-the-art context learning method Context-EEG [17] which incorporates the temporal features for the task of EEG seizure detection.

### Detection performance

We compare the seizure detection performance of our proposed model (WT-CtxFusionEEG) with the aforementioned baseline methods. We also implement a reduced model (WT-CtxEEG) that combines the previous ContextEEG method with our scalogram sequence representations. We summarize the testing results of seizure detection in Tables 2 and 3. We can observe that the overall performance of our proposed WT-CtxFusionEEG is better than the baselines in terms of all the six evaluation measurements.

**Table 2:** Detection performance comparisons on two benchmark datasets

	CHB-MIT Dataset				Bonn Dataset			
Method	Precision	Recall	F1-score	Accuracy	Precision	Recall	F1-score	Accuracy
PSVM	0.8887	0.8766	0.8826	0.8136	1.0000	0.5593	0.7174	0.6060
PNN	0.6460	0.8981	0.7515	0.6630	0.9640	0.6301	0.7621	0.6990
PSDAEs	0.6625	0.8988	0.7627	0.6750	0.9760	0.7625	0.8561	0.8360
Context-EEG [17]	0.7408	0.9560	0.8348	0.7687	0.9980	0.8559	0.9215	0.9150
WT-PSVM	0.8142	0.9200	0.8638	0.7976	1.0000	0.8993	0.9470	0.9440
WT-PNN	0.8192	0.9863	0.8950	0.8485	1.0000	0.9025	0.9488	0.9460
WT-PSDAEs	0.9027	0.9177	0.9101	0.8594	1.0000	0.9276	0.9625	0.9610
WT-CtxEEG	0.9166	0.9837	0.9490	0.9222	1.0000	0.9634	0.9814	0.9810
WT-CtxFusionEEG	0.9608	0.9865	0.9725	0.9571	1.0000	1.0000	1.0000	1.0000

**Table 3:** AUC of ROC and PR curves of each method on two benchmark datasets

	CHB-MIT Dataset		Bonn Dataset	
Method	AUC-ROC	AUC-PR	AUC-ROC	AUC-PR
PSVM	0.7283	0.6012	0.6987	0.8111
PNN	0.7754	0.5487	0.8548	0.8608
PSDAEs	0.7166	0.3679	0.9608	0.9620
Context-EEG [17]	0.8784	0.8304	0.9930	0.9925
WT-PSVM	0.8552	0.7411	0.9706	0.8603
WT-PNN	0.9432	0.7701	0.9696	0.9718
WT-PSDAEs	0.8214	0.7577	0.9779	0.9853
WT-CtxEEG	0.9782	0.9249	0.9994	0.9974
WT-CtxFusionEEG	0.9874	0.9649	1.0000	0.9980

From the given results, most methods on the CHB-MIT dataset perform worse than those on the Bonn dataset. This is because the rhythmic patterns in the multi-channel EEG records are less observable than those in the single-channel records. Although multiple channels can provide more information to describe EEG seizures, they also introduce high dimensions to data since some channels may be irrelevant and redundant to the seizure with different individuals [32]. Thus, most of the classifiers can easily extract distinct features benefiting from the simple patterns in frequency and amplitude on the Bonn dataset. In this situation, our WT-CtxFusionEEG method can achieve the best result of 100% in terms of F1-score and Accuracy.

Given the results of baselines, the NN-based models perform worse than the SVM-based models in the time domain, but achieve better in the time-frequency domain. It is because the raw biosignals contain noise that makes the neural network hard to reach a global minimum using gradient decent optimization algorithm. This observation can also be found from the performance comparison in different domains that most of the models take advantages of the EEG scalogram representation. We can justify that EEG seizure detector can capture more powerful information by incorporating handcrafted features. From the results, we can also observe that the performance of WT-PSDAEs, utilizing standard deep learning method, is better than WT-PNN and WT-PSVM. It results from the high-quality hidden features learned from the EEG scalograms. Regarding the context learning, both the Context-EEG and WT-CtxEEG models yield better results compared with the other corresponding baselines, respectively. The reason is that the temporal features extracted by such models help to enhance the feature representation. Furthermore, given the best result achieved by WT-CtxFusionEEG which adopts the strategy of integrated feature representation, we can conclude that our proposed model is able to capture representative features from EEG signals.

**Fig. 6:**
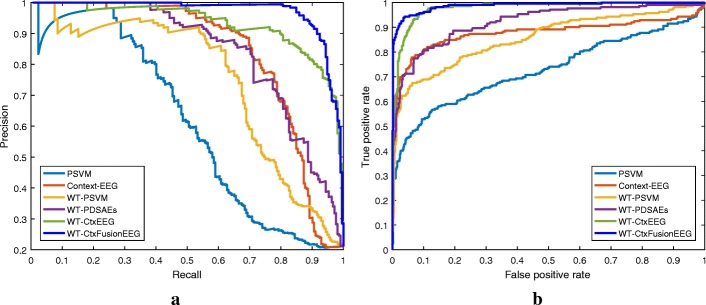
PR and ROC curves of the baselines and proposed methods on the CHB-MIT dataset. **a** plots the PR curves and **b** plots the ROC curves

Figure 6 illustrates the PR and ROC curves of each method on the CHB-MIT dataset, respectively. From the PR curves shown in Fig. 6a, we can see that the precision rate of the WT-CtxFusionEEG model decreases slowly at the beginning, which means that WT-CtxFusionEEG is able to obtain critical information to separate data effectively. This observation can also be found from the ROC curve of WT-CtxFusionEEG, where the true positive rate increases fast from the start, as shown in Fig. 6b. Moreover, according to the results listed in Table 3, the proposed WT-CtxFusionEEG method achieves the best AUC of 0.9649 and 0.9874 in terms of the PR and ROC, compared with the reduced model (WT-CtxEEG) with 0.9249 and 0.9782, respectively. Based on all the above analysis, we can conclude that our proposed WT-CtxFusionEEG approach can learn hidden representations in different aspects, and the multi-context fusion strategy provides complementary information towards each other, which is key for EEG seizure detection.

## Discussion

To further analyze the performance of our proposed WT-CtxFusionEEG approach, in this section, we conduct extensive experiments to discuss the effectiveness of our model.

### Parameter sensitivity analysis

**Fig. 7:**
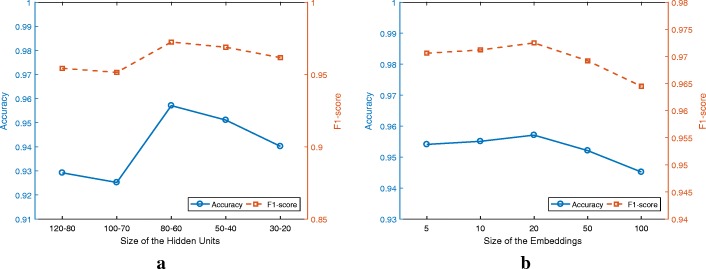
Performance variations with different parameter settings on the CHB-MIT dataset. **a** shows the sensitivity analysis with different sizes of hidden units, and **b** shows the sensitivity analysis with different sizes of embeddings

We conduct sensitivity analysis to discuss the impact of hyper-parameter configuration on the CHB-MIT dataset. Specifically, we study two main aspects that are the size of inherent units and the the size of embeddings, respectively. We plot the Accuracy and F1-score results using different settings of hyper-parameters, as shown in Fig. 7. Note that we use the aforementioned hyper-parameter setting as the basic configuration of our WT-CtxFusionEEG model. In each step, we vary one hyper-parameter while keeping others fixed to the basic configuration.

*Inherent unit size.* Fig. 7a shows the change of Accuracy and F1-score for different sizes of hidden units. From the figure, we can observe that the proposed model gets the best performance when the layer size is 80-60. We can also see that the dimension of hidden structure is reduced effectively and 80-60 is enough to capture the inherent features for each EEG channel. While too few hidden units would result in the proposed models being unable to learn enough features, too many hidden units would also put the proposed model at the risk of the curse of dimensionality.

*Embedding size.* We report the experimental results using different embedding sizes in Fig. 7b. From the figure, we can see that when the size of embedding vector is small, our model lacks the capability of capturing temporal features, resulting in limited performance on both Accuracy and F1-score. As we increase the size of embedding vector, our model shows an increasing modeling power. However, when the size is too large, we have insufficient samples to train the EEG embeddings, which results in a worse performance and stability. In our experiment, we choose 20 as the size of EEG embeddings.

In summary, despite of the influence, it is obvious that our proposed WT-CtxFusionEEG model consistently beat the baseline methods with different hyper-parameter settings.

### Wavelet comparative analysis

We discuss the performance influences of the proposed WT-CtxFusionEEG model using various mother wavelet functions, including the Morse, Bump, and Morlet wavelet. Table 4 lists the comparative performance under different mother wavelets based on the same parameter configuration on the CHB-MIT dataset. From the table, when the mother wavelet changes, our proposed WT-CtxFusionEEG model is stable and can still achieve comparable results. The comparison among wavelet functions shows that the Bump wavelet performs worse than the others. This is because the variance of Bump in frequency is relatively narrow, and the generated scalogram lacks to preserve detailed frequency information. The Morlet wavelet, adopting equal variance in time and frequency, performs the best, which demonstrates that the Morlet wavelet is more suitable for EEG seizure detection.

**Table 4:** Comparative performance of WT-CtxFusionEEG under different mother wavelets on the CHB-MIT dataset

Mother	AUC-ROC	AUC-PR	Precision	Recall	F1-score	Accuracy
Wavelet						
Morse	0.9543	0.9195	0.9279	0.9748	0.9508	0.9242
Bump	0.9642	0.9362	0.9039	0.9781	0.9396	0.9083
Morlet	0.9874	0.9649	0.9608	0.9865	0.9725	0.9571

## Conclusions

In this paper, we present and evaluate our proposed multi-context learning approach (WT-CtxFusionEEG) for automatic EEG seizure detection. The proposed approach is a multi-stage unsupervised feature learning model that explicitly takes into account the features extracted from three modules, including the global handcrafted engineering, channel-wise deep learning, and EEG embeddings. We transform EEG signals into time-frequency domain via wavelet transform, and generate the EEG scalogram sequence. We adopt GPCA to derive the global features from all-channel EEG scalograms in handcrafted feature space. The channel-wise inherent features are separately extracted from each EEG channel through SDAEs. We develop EEG embeddings to extract the temporal features with EEG semantic properties. To train the EEG seizure detector, the learned multi-context features are subsequently merged for classification. The effectiveness of the proposed method is evaluated on two benchmark biological datasets against several baselines. We empirically demonstrate that WT-CtxFusionEEG can learn representative features from different perspectives to better understand the characteristics of EEG seizure patterns.

## Abbreviations

AE: Autoencoder
AUC: Area under the curve
CWT: Continuous wavelet transform
DAE: Denoising autoencoder
EEG: Electroencephalogram
GPCA: Global principal component analysis
NN: Neural networks
PCA: Principal component analysis
PR: Precision recall
ROC: Receiver operator characteristic
SDAEs: Stacked denoising autoencoders
SVM: Support vector machine

## References

[1] Organization WH. Epilepsy Fact Sheet. 2017. http://www.who.int/mediacentre/factsheets/fs999/en/. Accessed 3 Jan 2017.

[2] Giannakakis G, Sakkalis V, Pediaditis M, Tsiknakis M (2015). Methods for seizure detection and prediction: an overview. Mod Electroencephalographic Assess Tech Theory Appl.

[3] Tong S, Thakor NV (2009). Quantitative EEG Analysis Methods and Clinical Applications.

[4] Fisher RS, Boas WvE, Blume W, Elger C, Genton P, Lee P, Engel J (2005). Epileptic seizures and epilepsy: definitions proposed by the international league against epilepsy (ilae) and the international bureau for epilepsy (ibe). Epilepsia.

[5] Yang G-Z, Yacoub M (2006). Body Sensor Networks vol. 1.

[6] Suo Q, Ma F, Yuan Y, Huai M, Zhong W, Gao J, Zhang A (2018). Deep patient similarity learning for personalized healthcare. IEEE Trans NanoBioscience.

[7] Ma F, Gao J, Suo Q, You Q, Zhou J, Zhang A (2018). Risk prediction on electronic health records with prior medical knowledge. Proceedings of the 24th ACM SIGKDD International Conference on Knowledge Discovery & Data Mining.

[8] Johnson AE, Ghassemi MM, Nemati S, Niehaus KE, Clifton DA, Clifford GD (2016). Machine learning and decision support in critical care. Proc IEEE.

[9] Mormann F, Andrzejak RG, Elger CE, Lehnertz K (2007). Seizure prediction: the long and winding road. Brain.

[10] Shin Y, Lee S, Ahn M, Cho H, Jun SC, Lee H-N (2015). Noise robustness analysis of sparse representation based classification method for non-stationary eeg signal classification. Biomed Signal Process Control.

[11] Acharya UR, Sree SV, Swapna G, Martis RJ, Suri JS (2013). Automated eeg analysis of epilepsy: a review. Knowl-Based Syst.

[12] Faust O, Acharya UR, Adeli H, Adeli A (2015). Wavelet-based eeg processing for computer-aided seizure detection and epilepsy diagnosis. Seizure.

[13] Mallat S (2008). A Wavelet Tour of Signal Processing: the Sparse Way.

[14] Längkvist M, Karlsson L, Loutfi A (2014). A review of unsupervised feature learning and deep learning for time-series modeling. Pattern Recogn Lett.

[15] Antoniades A, Spyrou L, Took CC, Sanei S (2016). Deep learning for epileptic intracranial eeg data. Machine Learning for Signal Processing (MLSP), 2016 IEEE 26th International Workshop On.

[16] Alotaiby TN, Alshebeili SA, Alshawi T, Ahmad I, El-Samie FEA (2014). Eeg seizure detection and prediction algorithms: a survey. EURASIP J Adv Signal Process.

[17] Xun G, Jia X, Zhang A (2016). Detecting epileptic seizures with electroencephalogram via a context-learning model. BMC Med Inform Decis Making.

[18] Li X, Jia X, Xun G, Zhang A (2015). Improving eeg feature learning via synchronized facial video. Big Data (Big Data), 2015 IEEE International Conference On.

[19] Yuan Y, Xun G, Jia K, Zhang A (2017). A novel wavelet-based model for eeg epileptic seizure detection using multi-context learning. Bioinformatics and Biomedicine (BIBM), 2017 IEEE International Conference On.

[20] Yuan Y, Xun G, Suo Q, Jia K, Zhang A. Wave2vec: Learning deep representations for biosignals. In: Data Mining (ICDM), 2017 IEEE International Conference On. IEEE: 2017. p. 1159–64.

[21] Cortes C, Vapnik V (1995). Support-vector networks. Mach Learn.

[22] Gotman J, Flanagan D, Zhang J, Rosenblatt B (1997). Automatic seizure detection in the newborn: methods and initial evaluation. Electroencephalogr Clin Neurophysiol.

[23] Wold S, Esbensen K, Geladi P (1987). Principal component analysis. Chemometr Intell Lab Syst.

[24] Vincent P, Larochelle H, Lajoie I, Bengio Y, Manzagol P-A (2010). Stacked denoising autoencoders: Learning useful representations in a deep network with a local denoising criterion. J Mach Learn Res.

[25] Vincent P, Larochelle H, Bengio Y, Manzagol P-A (2008). Extracting and composing robust features with denoising autoencoders. Proceedings of the 25th International Conference on Machine Learning.

[26] Hinton GE, Salakhutdinov RR (2006). Reducing the dimensionality of data with neural networks. Science.

[27] Bengio Y, Lamblin P, Popovici D, Larochelle H (2007). Greedy layer-wise training of deep networks. Adv Neural Inf Process Syst.

[28] Mikolov T, Chen K, Corrado G, Dean J. Efficient estimation of word representations in vector space. arXiv preprint arXiv:1301.3781. 2013.

[29] Shoeb AH. Application of machine learning to epileptic seizure onset detection and treatment. 2009. PhD thesis, Massachusetts Institute of Technology.

[30] Goldberger AL, Amaral LA, Glass L, Hausdorff JM, Ivanov PC, Mark RG, Mietus JE, Moody GB, Peng C-K, Stanley HE (2000). Physiobank, physiotoolkit, and physionet. Circulation.

[31] Andrzejak RG, Lehnertz K, Mormann F, Rieke C, David P, Elger CE (2001). Indications of nonlinear deterministic and finite-dimensional structures in time series of brain electrical activity: Dependence on recording region and brain state. Phys Rev E.

[32] Yuan Y, Xun G, Jia K, Zhang A (2017). A multi-view deep learning method for epileptic seizure detection using short-time fourier transform. Proceedings of the 8th ACM International Conference on Bioinformatics, Computational Biology, and Health Informatics.

[33] Yuan Y, Xun G, Ma F, Suo Q, Xue H, Jia K, Zhang A (2018). A novel channel-aware attention framework for multi-channel eeg seizure detection via multi-view deep learning. Biomedical & Health Informatics (BHI), 2018 IEEE EMBS International Conference On.

[34] McCulloch WS, Pitts W (1943). A logical calculus of the ideas immanent in nervous activity. Bull Math Biophys.

